# Toxicological Investigations on the Sea Urchin *Tripneustes gratilla* (*Toxopneustidae*, Echinoid) from Anaho Bay (Nuku Hiva, French Polynesia): Evidence for the Presence of Pacific Ciguatoxins

**DOI:** 10.3390/md16040122

**Published:** 2018-04-06

**Authors:** Hélène Taiana Darius, Mélanie Roué, Manoella Sibat, Jérôme Viallon, Clémence Mahana iti Gatti, Mark W. Vandersea, Patricia A. Tester, R. Wayne Litaker, Zouher Amzil, Philipp Hess, Mireille Chinain

**Affiliations:** 1 Institut Louis Malardé (ILM), Laboratory of Toxic Microalgae—UMR 241-EIO, PO Box 30, Papeete 98713, Tahiti, French Polynesia; jviallon@ilm.pf (J.V.); cgatti@ilm.pf (C.M.i.G.); mchinain@ilm.pf (M.C.); 2 Institut de Recherche pour le Développement (IRD)—UMR 241-EIO, PO Box 53267, Pirae 98716, Tahiti, French Polynesia; melanie.roue@ird.fr; 3 IFREMER, Phycotoxins Laboratory, F-44311 Nantes, France; manoella.sibat@ifremer.fr (M.S.); zouher.amzil@ifremer.fr (Z.A.); philipp.hess@ifremer.fr (P.H.); 4 National Oceanic and Atmospheric Administration, National Ocean Service, Centers for Coastal Ocean Science, Beaufort Laboratory, Beaufort, NC 28516, USA; mark.w.vandersea@noaa.gov (M.W.V.); wayne.litaker@noaa.gov (R.W.L.); 5 Ocean Tester, LLC, Beaufort, NC 28516, USA; ocean.tester@gmail.com

**Keywords:** ciguatera poisoning, ciguatoxins, *Tripneustes gratilla*, sea urchin, *Echinoidea*, *Gambierdiscus polynesiensis*, window screens, artificial substrates, qPCR assays, CBA-N2a, LC-MS/MS

## Abstract

The sea urchin *Tripneustes gratilla* (*Toxopneustidae*, Echinoids) is a source of protein for many islanders in the Indo-West Pacific. It was previously reported to occasionally cause ciguatera-like poisoning; however, the exact nature of the causative agent was not confirmed. In April and July 2015, ciguatera poisonings were reported following the consumption of *T. gratilla* in Anaho Bay (Nuku Hiva Island, Marquesas archipelago, French Polynesia). Patient symptomatology was recorded and sea urchin samples were collected from Anaho Bay in July 2015 and November 2016. Toxicity analysis using the neuroblastoma cell–based assay (CBA-N2a) detected the presence of ciguatoxins (CTXs) in *T.* *gratilla* samples. *Gambierdiscus* species were predominant in the benthic assemblages of Anaho Bay, and *G.* *polynesiensis* was highly prevalent in in vitro cultures according to qPCR results. Liquid chromatography–tandem mass spectrometry (LC-MS/MS) analyses revealed that P-CTX-3B was the major ciguatoxin congener in toxic sea urchin samples, followed by 51-OH-P-CTX-3C, P-CTX-3C, P-CTX-4A, and P-CTX-4B. Between July 2015 and November 2016, the toxin content in *T.* *gratilla* decreased, but was consistently above the safety limit allowed for human consumption. This study provides evidence of CTX bioaccumulation in *T.* *gratilla* as a cause of ciguatera-like poisoning associated with a documented symptomatology.

## 1. Introduction

**Figure 1:**
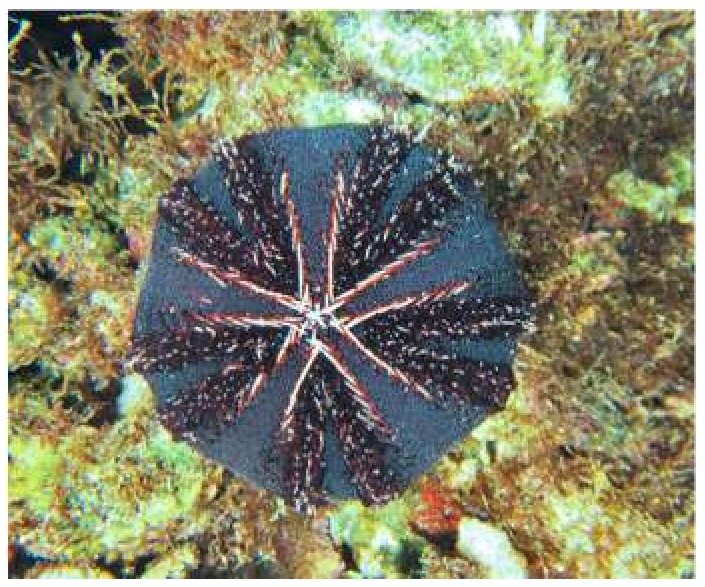
*Tripneustes gratilla* (Linnaeus, 1767) (photo credit: © ILM).

*Tripneustes gratilla* Linnaeus, 1758 (*Echinoidea*, *Temnopleuroida*, *Toxopneustidae*) (Figure 1) is a fast-growing echinoid found in isolated populations throughout the warm temperate Indo-Pacific region [1]. *T. gratilla* is commonly known as the “collector urchin,” as it tends to cover itself with debris (sand, rocks) for protection from sunlight and predators [2]. *Tripneustes* species are mostly grazers of sea grasses in tropical regions [3,4] and macroalgae in more temperate regions [1,5]. By nature, sea urchins must ingest and process large quantities of macroalgae and/or sea grass to meet their nutritional requirements for protein [6]. According to Byrne et al., 2008 [7], *Tripneustes* species are ecologically important, especially in sea grass habitats, and are often keystone species as primary herbivores. Related to this rapid consumption of algae and sea grasses, *T. gratilla* is sometimes considered a pest in seaweed farms in the Philippines [8]; however, this marine generalist herbivore has also been used as biocontrol for invasive algae [9].

This edible species produces high-quality gonads with excellent market value, and is one of the most commercially important sea urchin species in several countries, including Australia, Japan, the Philippines, and South Africa [5,10]. International demand for such high-quality urchin roe has, however, led to overfishing of natural populations of this and other echinoid species [10]. Apart from its importance in the international sea urchin industry, *T. gratilla* are also a highly prized delicacy and are part of the subsistence diet in Pacific Island countries and territories (PICTs) [11,12,13]. These echinoderms can be picked off the reef at low tide or collected by free diving in shallow waters, an activity mainly carried out by women and children [11,12,13]. 

**Figure 2:**
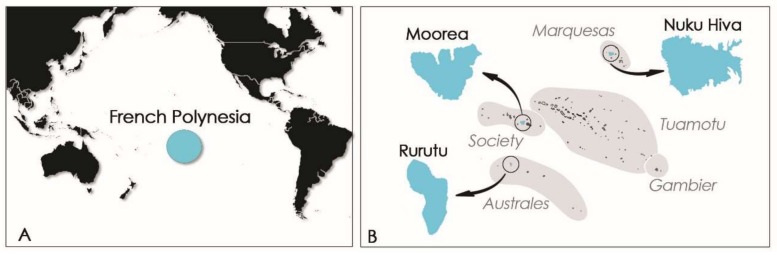
Maps of (**A**) French Polynesia and (**B**) Moorea Island (Society archipelago), Rurutu Island (Australes archipelago), and Nuku Hiva Island (Marquesas archipelago).

In French Polynesia, the local population is fond of sea urchins like *T. gratilla*, eating crude roe despite the toxic risk incurred. At the end of the 1950s, *T. gratilla* was known by the locals to be toxic in one part of the shore reef on the Island of Moorea (Society Island, French Polynesia; Figure 2) [14]. 

Years later, in 2009, its consumption led to the poisoning of six persons on the Island of Rurutu (Australes archipelago, French Polynesia; Figure 2B) [15]. The severe gastrointestinal and neurological symptoms exhibited by these patients were consistent with ciguatera fish poisoning (CFP) syndrome [15]. Following this toxic episode, Pawlowiez et al., 2013 [15] suggested the presence of ciguatoxin-like compounds in sea urchin specimens of Rurutu Island. Evidence to support this hypothesis was obtained by extracting *T. gratilla* tissues, fractionating the extracts by means of high-performance liquid chromatography (HPLC), and then assaying the fractions for toxicity using both the neuroblastoma cell–based (CBA-N2a) and radioactive receptor binding (_R_RBA) assays designed to detect ciguatoxins (CTXs). Certain fractions were shown to have activity consistent with CTXs. Known to be potent marine neurotoxins, CTXs are produced by the benthic dinoflagellate *Gambierdiscus* spp. and bioaccumulated in the food chain, classically in herbivorous, omnivorous, and carnivorous fishes [16,17]. However, in PICTs, marine invertebrates such as giant clams (e.g., *Tridacna maxima*, *Hippopus hippopus*) and trochus (*Tectus niloticus*) can also accumulate CTXs and are responsible for sporadic and severe poisoning incidents [15,18,19,20,21].

More recently, another ciguatera poisoning event involving three people was reported from Nuku Hiva Island (Marquesas archipelago, French Polynesia; Figure 2) following the consumption of *T. gratilla* collected from Anaho Bay in April and July 2015. These events prompted the collection of additional *T. gratilla* specimens from the toxic area in July 2015 and November 2016 for toxicological investigation. This paper describes both the symptomatology associated with this poisoning event and the toxicological analyses conducted on *T. gratilla* samples using CBA-N2a to search for the potential presence of CTX in samples. In addition, liquid chromatography coupled with tandem mass spectrometry (LC-MS/MS) was used for both identification of ciguatoxin analogs and multitoxin screening for a variety of other marine toxins: neurologic shellfish toxins (NSPs), i.e., brevetoxins (PbTX1 to PbTX10); paralytic shellfish poisoning (PSP) toxins, i.e., carbamates (STX, NEO-STX, GTX1-GTX4), *N*-sulfocarbamoyls (GTX5, GTX6, and C1 to C4), and decarbamoyls (dcSTX, dcNEO, dcGTX1-dcGTX4); diarrhetic shellfish poisoning (DSP) toxins, i.e., okadaic acid (OA), dinophysistoxins (DTXs), pectenotoxins (PTXs), azaspiracids (AZAs), and yessotoxins (YTXs); and cyclic imines (fast-action toxins (FATs)), i.e., gymnodimines (GYMs), spirolides (SPXs), and pinnatoxins (PnTXs). 

Finally, the abundance of *Gambierdiscus* species in Anaho Bay and the toxic potential of several *Gambierdiscus* clonal cultures established from the field-collected material were assessed using quantitative polymerase chain reaction (qPCR) assays and CBA-N2a to confirm whether they were the source of the ciguatoxins. 

## 2. Results

### 2.1. Patient Descriptions and Acute Clinical Manifestations

In 2015, two poisoning events occurred in a three-month interval between April and July, affecting a 57-year-old woman, a 62-year-old man, and a 65-year-old woman. All of them presented at the hospital on Nuku Hiva Island with a poisoning syndrome evocative of ciguatera poisoning (i.e., a combination of gastrointestinal, neurological, and general disorders) after consuming *T. gratilla* specimens. These three patients had eaten raw gonads of *T. gratilla* sprinkled with lemon juice. Clinical manifestations reported by physicians at the Nuku Hiva hospital (9 days postpoisoning for patients 1 and 2; 5 days postpoisoning for patient 3) are shown in Table 1.

As they lived in a secluded bay in the northern part of the island, and due to extreme tiredness and lack of strength, patients 1 and 2 waited 9 days and patient 3 waited 5 days postpoisoning before they were able to consult at the only hospital on the island, located in the south.

Based on medical reports, all patients first experienced nausea, vomiting, and diarrhea in the hour following *T. gratilla* consumption. These symptoms were associated with neurological manifestations such as cold allodynia (abnormal pain/discomfort in contact with cold stimuli), tingling of the extremities (hands, feet) and face, touch disturbances, itching, burning of the mouth and throat, headache, and dizziness. Among general symptoms, all patients presented with asthenia, myalgia, and urogenital discomfort, burning, or pain. Patients 1 and 2 experienced transient hypothermia with chills, while patients 1 and 3 developed vision disorder and arthralgia, respectively. No cardiovascular disorders were observed or might have been missed due to late presentation. One month postpoisoning, patients 1 and 2 still experienced extreme tiredness, digestive symptoms (constipation), headache, cold allodynia, and itching of the face.

### 2.2. Abundance of Gambierdiscus spp. in Anaho Bay

*Gambierdiscus* cells were collected from macroalgae and from six window screen (WS) artificial substrate sampling devices deployed at Anaho Bay on Nuku Hiva Island during a field mission in July 2015 (Table 2 and Table 3).

Microscopic cell enumeration performed on sample aliquots revealed that macroalgal substrates (namely turf-like and *Halimeda* sp.) were colonized by high numbers of *Gambierdiscus* cells (567 ± 546 cells/g of macroalgae) compared to *Ostreopsis* and *Prorocentrum*, whose cell densities were 17- and 189-fold lower, respectively (Table 2). The *Gambierdiscus* densities varied from 146 to 1625 cells/g of macroalgae, confirming the patchy distribution of *Gambierdiscus* on macroalgal substrates. Similar patchiness was observed on WS (artificial) substrates, with densities varying from 1225 to 38,300 cells/150 cm^2^. The coefficients of variation were higher when elevated cell densities were enumerated from both macroalgae and WSs, but were lower when low cell counts were obtained (Table 3). *Gambierdiscus* spp. were also the dominant taxa compared to co-occurring *Ostreopsis* and *Prorocentrum* species (cell densities two- to five-fold lower, respectively) on WS substrates. Overall, the artificial substrates consistently yielded higher cell densities than natural macroalgal substrates (Table 2). 

In parallel, qPCR assays using 10 distinct sets of *Gambierdiscus* species-specific primers were performed on WS samples to assess the relative abundance of *Gambierdiscus* species in Anaho Bay. Unfortunately, due to partial degradation of DNA in the samples, consistent cell enumeration could not be attained by qPCR. However, at least five *Gambierdiscus* species were detected in Anaho Bay: *Gambierdiscus carpenteri*, *G. polynesiensis*, *G. caribaeus*, *G. pacificus*, and *G. australes*. Additionally, qPCR assays were performed on eight clonal cultures of *Gambierdiscus* established from cells isolated from WS samples. The assays revealed that four strains belonged to *G. polynesiensis*, two strains to *G. carpenteri*, and two strains to *G. pacificus* (Table 3).

### 2.3. Toxicity Results Using CBA-N2a

#### 2.3.1. Toxicity of *Gambierdiscus* Clonal Cultures

**Figure 3:**
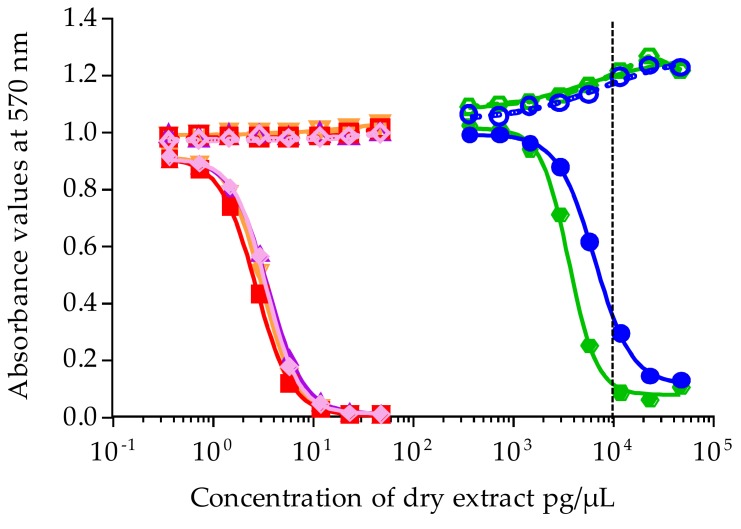
Dose-response curves of neuro-2a cells not treated with ouabain and veratridine (OV^−^; open symbols) and treated with ouabain and veratridine (OV^+^; solid symbols), when exposed to increasing concentrations of liposoluble fractions of *Gambierdiscus* strains isolated from Anaho Bay. Liposoluble fractions from NHA1 (**○/●**), NHA4 (◇/◆), NHA9 (□/■), NHA14 (△/▲), NHA22 (▽/▼), NHA24 (**○/●**). Data represent the mean ± SD of three independent experiments (each concentration run in triplicate). The dotted vertical line corresponds to the maximum concentration of dry extract (MCE = 10,000 pg/µL) for matrix interference.

Liposoluble fractions from clonal cultures of *Gambierdiscus* were tested by CBA-N2a under OV^+^ conditions (neuro-2a cells treated with ouabain and veratridine mixture) and OV^−^ conditions (untreated neuro-2a cells without ouabain and veratridine added) (see Section 4.3). Among eight clonal cultures tested, two strains of *G. carpenteri* (NHA19 and NAH20) were nontoxic (data not shown), whereas four strains of *G. polynesiensis* and two strains of *G. pacificus* displayed CTX-like toxic activity on neuroblastoma cells; i.e., all six liposoluble fractions showed no cytotoxicity under OV^−^ conditions versus a sigmoidal dose-response curve in OV^+^ conditions, a response typical of CTX bioactivity (Figure 3). 

Raw EC_50_ values for *Gambierdiscus* strains expressed in pg/µL of dry extract were further converted to cell equiv./µL. The EC_50_ converted ranged from (6.77 ± 0.38) × 10^−4^ to 2.66 ± 0.21 cell equiv./µL (Table 4). The mean toxin content calculated for three of the *G. polynesiensis* strains, NHA4, NHA14, and NHA22, were similar, at 1.61 ± 0.08, 1.68 ± 0.10, and 1.69 ± 0.06 pg P-CTX-3C equiv./cell (*n* = 3), respectively, and slightly higher for NHA9, at 2.13 ± 0.12 pg P-CTX-3C equiv./cell (*n* = 3) (Table 4). For the two strains of *G. pacificus*, NHA1 and NHA24, the mean toxin contents were (5.44 ± 0.42) × 10^−4^ and (11.11 ± 0.37) × 10^−4^ pg P-CTX-3C equiv./cell (*n* = 3), respectively (Table 4). When comparing the toxin content among these six strains, *G. polynesiensis* strains were 1450- to 3922-fold more toxic than *G. pacificus* strains.

#### 2.3.2. Toxicity of *Tripneustes gratilla* Samples

The toxicity of *T. gratilla* extracts prepared from field samples collected from Anaho Bay in July 2015 and November 2016 was investigated by testing both liposoluble fractions (LF70/30, LF90/10, LF100) and hydrosoluble fractions (HF50/50, HF70/30, HF90/10, HF100). 

**Figure 4:**
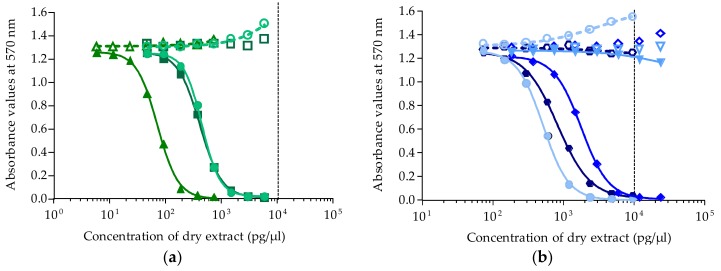
Dose-response curves of neuro-2a cells in OV^−^ (open symbols) and OV^+^ (solid symbols) conditions, when exposed to increasing concentrations of *Tripneustes gratilla* fractions (Anaho Bay, July 2015). (**a**) Liposoluble fractions LF70/30 (**○/●)**, LF90/10 (△/▲) and LF100 (□/■); (**b**) hydrosoluble fractions HF50/50 (▽/▼), HF70/30 (◇/◆), HF90/10 (**○/●**), and HF100 (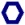/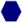). Data represent the mean ± SD of two independent experiments (each concentration run in triplicate). The dotted vertical line corresponds to the maximum concentration of dry extract (MCE = 10,000 pg/µL) for matrix interferences.

Except for fraction HF50/50, all *T. gratilla* fractions from samples collected in July 2015 displayed a negative response in OV^−^ conditions and a typical sigmoidal dose-response curve in OV^+^ conditions, a result characteristic of the mode of action of CTXs (Figure 4a,b). 

For all fractions that tested positive by CBA-N2a, raw EC_50_ values expressed in pg/µL of dry extract were further converted to µg tissue equiv./µL. For liposoluble fractions, the EC_50_ values were 3.92 ± 0.77 (*n* = 2), 0.08 ± 0.01 (*n* = 4), and 1.06 ± 0.19 (*n* = 4) µg tissue equiv./µL for LF70/30, LF90/10, and LF100, respectively, with LF90/10 being the most potent fraction (Figure 4a). For hydrosoluble fractions, EC_50_ values estimated for HF70/30, HF90/10, and H100 were 14.26 ± 3.48 (*n* = 4), 2.10 ± 0.68 (*n* = 3), and 6.49 ± 1.66 (*n* = 4) µg tissue equiv./µL, respectively, with HF90/10 being the most potent hydrosoluble fraction (Figure 4b). 

**Figure 5:**
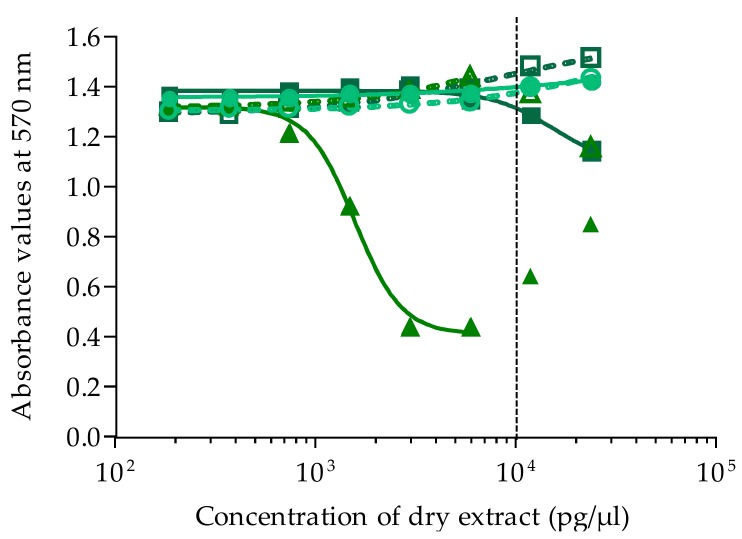
Dose-response curves of neuro-2a cells in OV^−^ (open symbols) and OV^+^ (solid symbols) conditions, when exposed to increasing concentrations of *Tripneustes gratilla* fractions (Anaho Bay, November 2016). Liposoluble fractions LF70/30 (**○/●**), LF90/10 (△/▲), and LF100 (□/■). Data represent the mean ± SD of two independent experiments (each concentration run in triplicate). The two last points of LF90/10 in OV^−^ and OV^+^ conditions were not included in the curve, as they increased and were above the MCE. The dotted vertical line corresponds to the maximum concentration of dry extract (MCE = 10,000 pg/µL) for matrix interferences.

As for *T. gratilla* samples collected in November 2016, a response typical of P-CTXs was observed only in fraction LF90/10 (EC_50_ = 4.67 ± 1.00 µg tissue equiv./µL, *n* = 2), whereas all other fractions (LF70/30, LF100, HF50/50, HF70/30, HF90/10, HF100) were found to be nontoxic (Figure 5). 

These EC_50_ values were then used to infer the mean toxin content in the positive fractions of *T. gratilla* samples (Table 5). Regardless of the sampling date, ≅97% of the total toxin content was found in the liposoluble fractions, primarily in LF90/10 (88%), followed by LF100 (7%), and only 3% in hydrosoluble fractions. Additionally, while CTXs were detected in almost all fractions collected in July 2015, only one fraction was found to contain residual ciguatoxicity in November 2016. The total toxin content estimates in *T. gratilla* samples collected from Anaho Bay reached 20.19 ± 2.96 ng P-CTX-3C equiv./g of tissue in July 2015, then dropped to 0.32 ± 0.07 ng P-CTX-3C equiv./g of tissue in November 2016, corresponding to a 64-fold decrease in toxicity (Table 5).

### 2.4. Characterization of Toxin Profiles in Tripneustes gratilla Toxic Fractions Using LC-MS/MS

**Figure 6:**
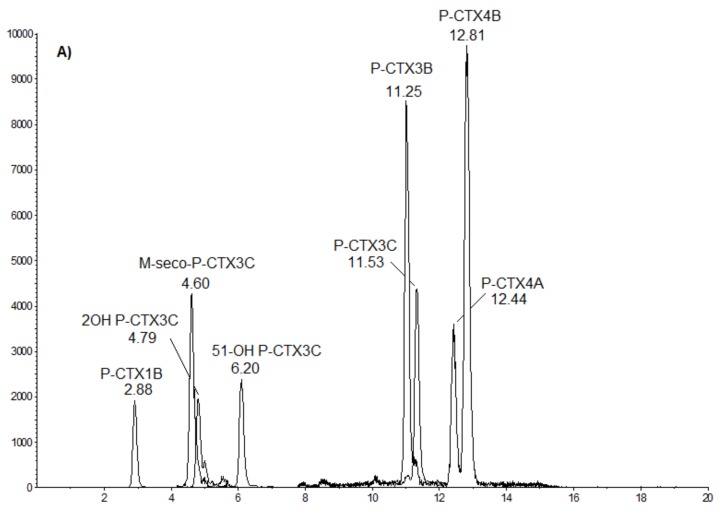

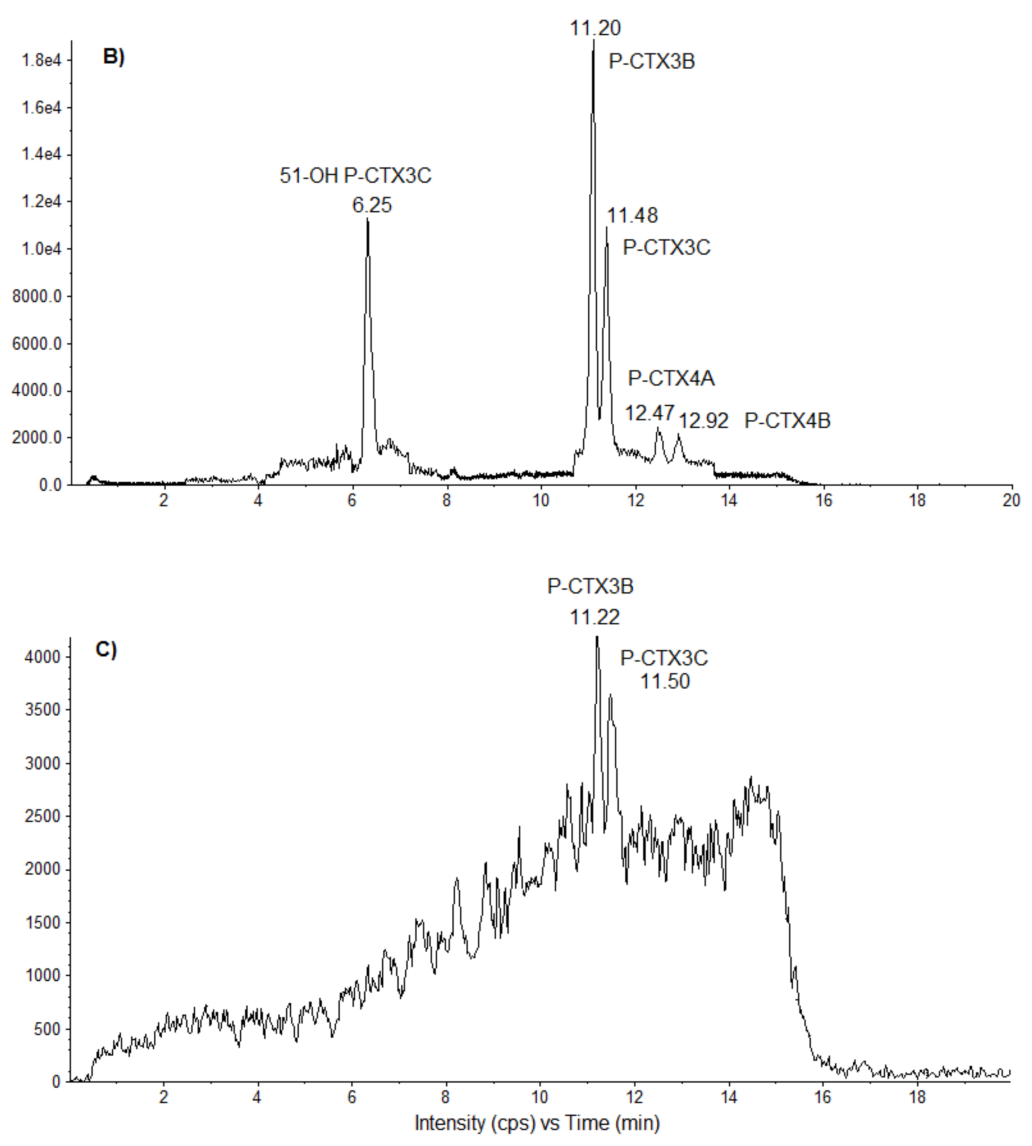
LC-MS/MS chromatograms obtained for (**A**) a mix of P-CTX standards (provided by Institut Louis Malardé) and solid phase extraction–purified extracts of *Tripneustes gratilla* collected from Anaho Bay in (**B**) July 2015 and (**C**) November 2016. Chromatograms were acquired following the procedure described in Section 4.5, in positive multireaction monitoring mode.

LC-MS/MS analyses confirmed the presence of P-CTXs in toxic fractions of *T. gratilla* collected from Anaho Bay in July 2015 and November 2016 (Figure 6A–C). 

In *T. gratilla* samples collected in July 2015 (Figure 6B), P-CTX-3B, P-CTX-3C, 51-OH-P-CTX-3C, P-CTX-4A, and P-CTX-4B were formally identified by comparison with the corresponding standards (Figure 6A). For the November 2016 samples, only P-CTX-3B and P-CTX-3C were identified, albeit at concentrations close to the detection limit, whereas 51-OH-P-CTX-3C, P-CTX-4A, and P-CTX-4B were not detected (Figure 6C). 

In addition, the relative concentration of each of these congeners in *T. gratilla* toxin extracts was assessed using a calibration range of P-CTX-3C (Wako supplier) (Table 6). In the solid phase extraction (SPE Si) extract obtained from *T. gratilla* collected in July 2015, P-CTX-3B appeared to be the most prevalent toxin congener (44%), followed by 51-OH-P-CTX-3C (25%), P-CTX-3C (20%), P-CTX-4A (5%), and P-CTX-4B (5%), whereas the toxin profile in the November 2016 samples was composed of 58% P-CTX-3C and 42% P-CTX-3B.

Based on LC-MS/MS analysis, the total toxin contents in *T. gratilla* samples from July 2015 and November 2016 were estimated at 21.5 and 1.48 ng P-CTX-3C equiv./g of tissue, respectively, corresponding to a 15-fold decrease in toxicity between these two sampling periods (Table 6). 

The multitoxin screening conducted on the *T. gratilla* samples from Anaho Bay (July 2015 and November 2016) showed the absence of NSP toxins (brevetoxin group), DSP toxins as okadaic acid (OA), dinophysistoxins (DTXs), azaspiracids (AZAs), and yessotoxins (YTXs), no pectenotoxins (PTXs), and FATs comprising gymnodimines (GYMs), spirolides (SPXs), and pinnatoxins (PnTXs).

## 3. Discussion

For Pacific Islanders, not only fish, but also marine invertebrates are a valuable source of protein and revenue. Their consumption, however, may pose health hazards as a result of the bioaccumulation of potent marine biotoxins in marine food webs. In April and July 2015, two consecutive poisoning events involving three persons who developed a ciguatera-like syndrome after consuming *Tripneustes gratilla* specimens collected from the island of Nuku Hiva (Marquesas archipelago, French Polynesia) prompted field investigations and a follow-up study to better characterize the clinical picture of this uncommon poisoning and the toxins involved. 

In the April and July toxicity events, the three individuals promptly developed a wide array of digestive and neurological symptoms (including cold allodynia) following the consumption of raw gonads of *T. gratilla* (four urchins per person for patients 1 and 2, unknown for patient 3). The combination of gastrointestinal and neurological manifestations was a strong indication of ciguatera poisoning [22,23], except that the symptoms appeared very quickly, i.e., less than 1 h after the toxic meal. Among other neurological manifestations, hypothermia and chills were reported in all patients. While this response is still poorly documented to date, it may correspond to a biological response of the thermoregulatory system to CTX exposure [24,25]. Moreover, all patients described urogenital disturbances (itching, burning sensation, or pain), which has been suggested to be a side effect of the excretion of CTXs, as they are eliminated in urine and feces [26]. One month after the initial poisoning, patients 1 and 2 continued to manifest significant neurological symptoms. In a significant proportion of ciguatera patients, chronic, less intense symptoms can persist for months or years. More intense relapses may also occur [22]. 

The *T. gratilla* specimens consumed by the patients were not available for analysis. However, the mean toxin content of *T. gratilla* specimens collected in July 2015 was ~20 ng P-CTX-3C equiv./g of tissue. This was well above the advisory safety limit allowed by the U.S. Food and Drug Administration (FDA) and the European Food Safety Authority (EFSA), i.e., 0.01 ng P-CTX-1B equiv./g of tissue [27,28]. These guidance levels were set based on a 10-fold reduction of the lowest concentration of CTXs found in meal remnants known to cause human illness. Interestingly, similar digestive and neurological manifestations were also observed in nine people following consumption of the gastropod *Tectus niloticus* collected in the same area one year earlier [29,30]. In both cases, the onset of symptoms was very rapid: 1 h with *T. gratilla* and 2 to 4 h with *T. niloticus*. Interestingly, the P-CTX toxin profiles evidenced in *T. gratilla* and *T. niloticus* identified the same congeners in both toxic extracts, i.e., P-CTX-3B, P-CTX-3C, P-CTX-4A, and P-CTX-4B, except that 51-OH-P-CTX-3C was present only in *T. gratilla* specimens. The similarity in the toxin profiles further suggests common origin(s) for the toxins.

A common belief in ciguatera disease research is that cardiovascular symptoms are usually a sign of the severity of the poisoning, which is under the control of various factors, including the level of toxin content in toxic meals and the type of tissue ingested [31,32]. This assumption could not be linked to the present study because of unavailable information on cardiovascular disorders in these three patients and the possible concurrent use of alcohol. It is well known that in fish, toxins may be most concentrated in the head, viscera, and eggs and these should be avoided, as they often contain much higher CTX concentrations compared to flesh [22,33,34]. While sea urchin lovers would particularly eat the gonads, estimating the ingested dose was not feasible in this study, as gonad weight and gonad index were unknown. Moreover, it should be noted that this study did not address any P-CTX distribution among the different tissues of *T. gratilla*. This needs to be addressed in future research. 

CTXs are known to preferentially bind to site 5 of the alpha subunit of voltage-gated sodium channels (VGSCs), resulting in various perturbations of the biophysical properties of these channels, such as inhibition of part of their inactivation and a shift of their activation toward more negative potential [35,36]. In addition, ciguatoxins target and block voltage-dependent potassium (K^+^) channels in excitable membranes. These modifications of Na^+^ and K^+^ voltage-gated channel properties result in a variety of neurocellular perturbations, such as massive Na^+^ entry, increased intracellular Ca^2+^, enhanced neuronal excitability, sustained increase in spontaneous and transient increase in evoked neurotransmitter release from the motor nerve terminals, and uncoordinated muscular fiber contraction [37,38]. Based on a previous study [21], these algal CTX congeners show different potencies and polarities, as evidenced by CBA-N2a and LC-MS/MS experiments. P-CTX-3C and P-CTX-3B have the greatest adverse impact on the function of VGSCs, contrary to P-CTX-4A and P-CTX-4B, which are less potent, and also the less polar congeners as shown in the LC-MS/MS chromatograms. In contrast, Schlumberger et al., 2010 [37] showed that P-CTX-4B was about four times more effective than P-CTX-1B in affecting K^+^ channels critical to cardiac function [37]. The interaction between CTXs and ion channels, however, does not fully account for all the symptoms associated with ciguatera fish poisoning [36]. Other factors, such as individual susceptibility linked to a patient’s medical history or the human leucocyte antigen (HLA) genetic profile [39,40,41], are also important to explain the differences in clinical outcomes, and account for why the severity, diversity, and duration of symptoms cannot be linked solely to the toxin content and/or toxin profile of the seafood ingested.

The toxin contents estimated from CBA-N2a and LC-MS/MS data yielded quite similar results despite the use of two different chemical extraction protocols. A good congruence between these two different methods was noted by previous studies [42,43]. Our findings indicate that (i) *T. gratilla* were able to accumulate and retain CTXs in their tissues at levels well above the threshold for human poisoning and (ii) the general trend observed in the decrease of *T. gratilla* toxicity over time from Anaho Bay remains consistent between these two analytical methods. Such findings suggest that *T. gratilla* is able to naturally bioaccumulate P-CTXs in its tissues, and this should be regarded as a novel bioaccumulation pathway for ciguatera toxins in marine food webs.

As previously shown in the giant clam *Tridacna maxima* experimentally fed *G. polynesiensis* cells [44] and in the trochus *Tectus niloticus* [21], the toxin profile of the sea urchin *T. gratilla* mainly comprised four CTX congeners: P-CTX-3C, -3B, -4A, and -4B, with P-CTX-3B as the predominant congener, suggesting an enhanced bioaccumulation capacity for this analog in these marine invertebrates. These four analogs are known to be preferentially produced in *Gambierdiscus* cultures [45], suggesting that *Gambierdiscus* is a likely source of CTXs detected in *T. gratilla* from Anaho Bay. Indeed, collector urchins graze on just about any alga or sea grass available, although some urchins showed dietary preferences in laboratory studies [9]. In this process, *Gambierdiscus* cells sheltered in macroalgae could be easily ingested by this generalist herbivore. Other marine echinoids in the Dendrasteridae and Parechinidae families are able to bioaccumulate ASP toxins or palytoxins (PLTXs), such as *Dendraster exentricus* in California (USA) and *Paracentrotus lividus* in the south of France [46,47,48].

Regarding the abundance and species diversity of *Gambierdiscus* in the benthic assemblages of Anaho Bay, *Gambierdiscus* clearly dominated over other genera, such as *Ostreopsis* and *Prorocentrum*, in both natural (macroalgae) and artificial (WS devices) substrates monitored in our study. *Ostreopsis* and *Prorocentrum* are commonly found in association with *Gambierdiscus* as part of a mixed assemblage of benthic dinoflagellates in French Polynesian ciguateric biotopes [15,19,49,50]. Field observations also suggest that *Gambierdiscus* has been established for a long time in Anaho Bay, since two toxic *Gambierdiscus* blooms were previously described in this area back in 2004 [50], and the presence of this dinoflagellate in the same site was recently documented by Darius et al., 2018 [21]. *Gambierdiscus* cell abundance was consistently higher in WS samples than on macroalgae, as assessed by microscopic enumeration. Caution should be taken when interpreting these data gathered from artificial substrate deployments [51], but care also needs to be taken when macrophytes are collected, as these can have very different morphologies, which in turn affects the number of cells/g wet weight algae [52]. The advantage of using artificial substrates is the ability to collect clean samples for molecular identification purposes, as compared to strong inhibition encountered from collection on macroalgal samples [21,53,54]. As *Gambierdiscus* cells often move around, screens left out for 24 h allow an integrated measure of the cells present on the diverse substrates in the environment. Taken together, both methods showed high variability of cell densities, indicating small-scale patchy distribution of *Gambierdiscus* cells [49,52,55]. Overall, *Gambierdiscus* cell densities on macroalgae were 378-fold higher in Anaho Bay in July 2015 than in November 2016 [21].

Based on our qPCR data, at least five different species of *Gambierdiscus* were present in Anaho Bay. Unfortunately, reliable qPCR estimates of the relative abundance of these five species in WS samples could not be achieved in the present study. However, attempts to establish in vitro cultures of *Gambierdiscus* from cell isolates collected from field samples eventually yielded six distinct clonal cultures in the laboratory, with *G. polynesiensis* as the dominant species, a result consistent with field observations conducted in the same area in November 2016 [21]. *G. polynesiensis* is known as one of the most toxic species of *Gambierdiscus* described to date [56] and is believed to be a major contributor to the overall toxin flux into the food web in ciguateric areas. In other words, environments highly colonized by this species are more likely to foster ciguatoxic fish and marine products in general [45,56,57,58,59,60]. 

The toxicity of *T. gratilla* samples from Anaho Bay was monitored at two periods, 3 and 16 months after the poisoning events. The total toxin content estimated in toxic fractions based on CBA-N2a and LC-MS/MS data showed a 64- and 15-fold decrease, respectively, in the overall ciguatoxicity of these samples between July 2015 and November 2016. The same trend was observed in *T. niloticus*, suggesting a slow depuration rate for CTXs in these marine invertebrates [21]. However, this finding may also be confounded by additional accumulation of CTXs over the two-year period, even though *Gambierdiscus* abundance was low. It is important to stress that mechanisms controlling the uptake, metabolization, and depuration of CTXs are still poorly understood in marine invertebrate species prone to ciguatera, most notably echinoids and gastropods. Further investigation in this field of research will greatly benefit both ciguatera risk management programs and predictive models of CTX accumulation in benthic marine food webs.

The possible implication of echinoid and gastropod invertebrates in ciguatera events was suspected by Randall 1958 [14], who reported a toxic event following the consumption of *T. gratilla* in Moorea (Society Island, French Polynesia). Poisoning cases involving other marine invertebrates, *Tectus niloticus* (gastropod) and *Tridacna maxima* (bivalve mollusk), were implicated in toxic episodes reported in French Polynesia, New Caledonia, and the Cook Islands [15,18,20,29,30]. Starfishes *Ophidiaster ophidianus* and *Marthasterias glacialis* (phylum Echinodermata) collected from the northwestern Moroccan coast also have been reported to contain ciguatoxin congeners [61]. With regard to crustaceans and cephalopods, reports of toxic lobster *Panulirus penicillatus* and octopus have been described in the Republic of Kiribati and the Cook Islands [20,62]. In the case of toxic outbreaks caused by the consumption of giant clams, some authors have speculated on the possible contribution of other groups of marine organisms and toxins in atypical forms of ciguatera poisoning. In particular, the production of a suite of toxic metabolites, including CTX-like compounds, anatoxin-a and homoanatoxin-a, palytoxin, etc., by marine cyanobacteria in the Oscillatoriales group, has been documented in previous studies [18,63,64,65,66]. In the present effort, the multitoxin screening of *T. gratilla* for toxic extracts was negative for a variety of other toxic metabolites, including PSP and DSP toxins.

The high species diversity of the *Gambierdiscus* populations, including the species *G. polynesiensis*, a well-known toxin producer and source of CTXs in marine food webs, together with the high CTX content found in sea urchins, trochus [21], and fish [50] establish Anaho Bay as a ciguatera hotspot on Nuku Hiva Island. 

In conclusion, *T. gratilla* is of high value for the sea urchin industry as well as for many island communities in PICTs, where it represents a valuable nutritional resource. This study provides evidence of the presence of CTXs in *T. gratilla* associated with a documented symptomatology. It was unknown whether the whole tissue was extracted and what the distribution of P-CTXs was between different organs or tissues. Independent of this, it is clear that consumption of gonads of sea urchins constitutes a significant risk in areas prone to ciguatera. These novel findings also underline the importance of maintaining ecotoxicological surveillance in Anaho Bay, as well as the need for educational interventions among both local populations and visitors to increase public awareness of the toxic risk associated with ingestion of this highly prized lagoon resource in French Polynesia.

## 4. Materials and Methods

### 4.1. Acute Phase Clinical Description

The clinical description of acute manifestations of the poisoning was based on data collected from standardized CFP declaration forms, jointly developed by Institut Louis Malardé (Papeete, Tahiti) and the Public Health Directorate of French Polynesia in the frame of the CFP Epidemiological Surveillance Network ongoing in French Polynesia since 2007. As part of this surveillance program, all public health medical professionals are invited to report each diagnosed or suspected CFP case (code T61.0 according to the International Statistical Classification of Diseases and Related Health Problems, 10th Revision (ICD-10)). This anonymous clinical form provides information about the date of poisoning, the collection and consumption of the marine product, species identification, parts consumed, fishing/collection location, incubation time, cardiovascular constants, symptoms developed during the acute phase of the poisoning, and any additional information that physicians consider relevant.

### 4.2. Study Sites

**Figure 7:**
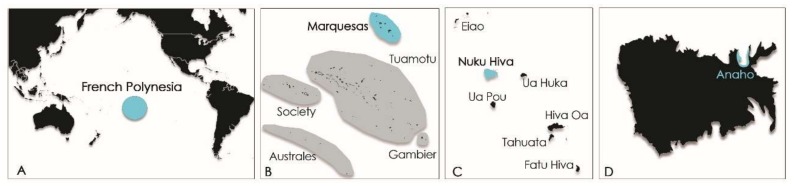
Maps of (**A**,**B**) French Polynesia, (**C**) Marquesas archipelago, and (**D**) study site of Anaho Bay on Nuku Hiva Island.

Samples (i.e., macroalgal substrates, window screens, and *T. gratilla* specimens) were collected from Anaho Bay, located on the northern coast of Nuku Hiva Island (Marquesas archipelago, French Polynesia) (Figure 7). Anaho Bay has been regarded as a long-standing hotspot of ciguatera since 2004 [50].

### 4.3. Biological Material and Sampling Procedures

#### 4.3.1. *Gambierdiscus* Samples and qPCR Assays

Collection of wild samples of *Gambierdiscus* using both the natural (i.e., macroalgae) and artificial (i.e., window screen, WS) substrate methods were conducted from Anaho Bay in July 2015 [67]. Briefly, ≅200 g of turf-like and *Halimeda micronesia* macroalgal hosts were collected at water depths of 1–5 m and examined for the presence of *Gambierdiscus* cells. Macroalgal samples were sealed within plastic bags underwater and shaken and kneaded vigorously to dislodge dinoflagellate cells. The detrital suspension was then successively filtered through 125, 40, and 20 µm mesh sieves and the 40 and 20 µm fractions were preserved in 50 mL of 5% formalin-seawater. Cell densities were assessed microscopically from 100 µL aliquots of the 2 fractions. Values are expressed as cells/g algal wet weight and represent the mean number of cells enumerated on n = 2–8 subsamples of the same host algae species [19]. The artificial substrate method used 150 cm^2^ WS devices assembled and deployed in the same areas. A total of 6 WSs were deployed per area for a 24 h period. After 24 h, WS were collected with 250 mL of ambient sea water and shaken to dislodge the cells. The entire volume was filtered through 10 μm polycarbonate filters that were replaced as the filters became obstructed. Then all filters used to process individual samples were transferred to 15 mL tubes with 8 mL of sterile filtered sea water. Before removing the filters, the tubes were shaken to dislodge *Gambierdiscus* cells. Enumeration of cells was performed microscopically on 1 mL aliquots and by qPCR estimation on the remaining 7 mL as described below. Cell concentrations are expressed as cells/150 cm^2^ [67].

Concurrently, 8 in vitro clonal cultures of *Gambierdiscus* spp. were established in the laboratory from single-cell isolates collected from WS samples, according to the method used by Chinain et al., 2010 [19]. 

Semiquantitative, species-specific qPCR assays were performed to survey the WS samples for relative cell abundance and *Gambierdiscus* species distribution. Each 7 mL WS aliquot was filtered onto 47 mm diameter, 8 µm pore size polycarbonate filters, and DNA was extracted from each filter as described by Vandersea et al., 2012 [68] using the Power Soil DNA isolation Kit (Qiagen, Hilden, Germany) following the manufacturer’s protocol, except 350 µL of cell lysate rather than the prescribed 450 µL was processed. The DNA extracts were eluted from the mini-columns using 50 µL of elution buffer and stored at 4 °C. Taxonomic identification of in vitro cultures and enumeration of wild cells collected on WS were conducted using species-specific qPCR assays described by Vandersea et al., 2012 [68] and Darius et al., 2018 [21] for *G. belizeanus*, *G. caribaeus*, *G. carpenteri*, *G. carolinianus*, *G. ruetzleri*, and *Gambierdiscus* ribotype 2 as well as 4 species currently reported in French Polynesia: *G. polynesiensis*, *G. toxicus*, *G. pacificus*, and *G. australes*.

PCR assays were performed using an Eppendorf Mastercycler^®^ ep RealPlex 4 system with white Eppendorf real-time tube strips (Eppendorf North America, Inc., Westbury, NY, USA) and a total reaction volume of 10.5 µL per tube. Each PCR reaction mixture contained 4.5 µL of 5 Prime RealMasterMix SYBR ROX 2.5× (0.05 units/µL Taq DNA polymerase, 10 mM Mg(CH_3_COO)_2_, 1.0 mM dNTPs, 20X SYBR^®^ Green solution), each primer at a concentration of 0.15 µM, 4.7µL of sterile deionized water, and 1 µL of template DNA. Thermal cycling conditions included denaturation at 95 °C for 2 min followed by 40 cycles at 95 °C for 10 s, annealing at 60 °C for 15 s, with a subsequent extension at 68 °C for 20 s. The fluorescence threshold was determined by the Eppendorf RealPlex 4 analytical software, and the PCR cycle during which fluorescence crossed the threshold was designated the quantification cycle (Cq). A melting curve analysis was performed following thermal cycling to check the specificity of the PCR reactions. The melting curve profile consisted of denaturation at 95 °C for 15 s followed by an annealing step at 60 °C for 15 s. The fluorescence was continuously monitored during a continuous 20 min temperature ramp from 60–95 °C, which was held at 95 °C for 15 s. The melting curve analysis was conducted by comparing the melting temperature peak of positive control DNA to other experimental DNA samples. A limit of ±0.5 °C for melting temperature peak shift was set as the cutoff for species-specific amplifications. The same procedure was followed for identification at the species level of the 8 clonal cultures of *Gambierdiscus* established from field samples.

#### 4.3.2. *Tripneustes gratilla* Samples

Two sampling campaigns were conducted in July 2015 and November 2016 in Anaho Bay (Figure 6), 3 and 16 months after the initial poisoning event. A total of 56 specimens of *T. gratilla* were collected, weighed, and measured individually, except for samples collected in July 2015, for which only min-max diameter values were available. Their weights were estimated by doing a mean of sample weights collected in November 2016 sharing the same diameter. Then, all the specimens from the same sampling date were pooled and freeze-dried prior to extraction (Table 7).

### 4.4. Extraction Procedures

Whole tissues of *T. gratilla* samples were extracted following the protocol from Darius et al., 2018 [21]. Briefly, each pool of *T. gratilla* specimens was extracted twice in methanol (MeOH) and twice in 50% aqueous MeOH, under sonication for 4 h. After 1 night at −20 °C, the crude extracts were centrifuged and the supernatants were pooled and dried under vacuum. The resulting crude extract was further partitioned between dichloromethane (CH_2_Cl_2_) and 60% aqueous MeOH (= hydrosoluble fraction, HF). The resulting CH_2_Cl_2_ phase was dried under vacuum and further defatted by a second solvent partition using cyclohexane and 80% aqueous MeOH (= liposoluble fraction, LF). The 60% aqueous MeOH phase (HF) and 80% aqueous MeOH phase (LF) were then evaporated and further purified on Sep-Pak C_18_ cartridges (360 mg sorbent per cartridge; Waters^®^, Saint-Quentin, France). For HF, the columns were preconditioned with 30% aqueous MeOH before loading extracts, washed with 30% aqueous methanol, and then eluted successively with 50%, 70%, and 90% aqueous methanol and pure methanol, resulting in 4 distinct hydrosoluble fractions: HF50/50, HF70/30, HF90/10, and HF100, respectively. For LF, the columns were preconditioned with 70% aqueous MeOH before loading extracts, washed with 70% aqueous methanol, and eluted successively with 90% aqueous methanol and pure methanol, leading to 3 distinct liposoluble fractions: LF70/30, LF90/10, and LF100. All these fractions were then dried in a SpeedVac concentrator, weighed, and stored at +4 °C until being tested for their toxicity. 

For *Gambierdiscus* spp. culture samples, only the dichloromethane phase in which lipid-soluble toxins such as CTXs are recovered was kept, dried, and stored until being tested for toxicity, as described by Chinain et al., 2010 [19].

### 4.5. Cell-Based Assay Using Neuroblastoma Cells

*Gambierdiscus* spp. and *T. gratilla* extracts were analyzed for their toxicity using the neuroblastoma cell-based assay (CBA-N2a), a test designed to detect the presence of toxins acting on voltage-gated sodium channels (VGSCs) such as brevetoxins and CTXs, which are both VGSC activators [69].

The procedure for CBA-N2a followed the method previously described by Roué et al., 2016 [44]. Briefly, a density of 45,000 neuroblastoma (neuro-2a) cells/200 µL/well in 5% fetal bovine serum (FBS) RPMI-1640 supplemented medium was seeded in a 96-well microtiter plate. After 20 to 24 h of growth at 37 °C in a humidified 5% CO_2_ atmosphere, all wells reached 100% confluence. Then, the medium was replaced by 200 µL of FBS 2.5% RPMI-1640 for half of the wells and 200 µL of the same medium containing an ouabain-veratridine (OV) solution at a concentration of 100/10 µM for the other half of the wells. 

Untreated cells without ouabain and veratridine added (OV^−^ conditions) or treated cells with the ouabain and veratridine mixture (OV^+^ conditions) were first exposed to P-CTX-3C, P-CTX-3B, P-CTX-4A, P-CTX-4B, and P-CTX-1B using the same calibration as performed in Darius et al., 2018 [21]. The P-CTXs used in this study were from the Institut Louis Malardé bank of standards.

The maximum concentration of dry extract (MCE) that does not induce unspecific mortality in neuro-2a cells not exposed to OV was established at 10,000 pg/µL for both *Gambierdiscus* and sea urchin matrices, whatever the fraction. All extracts were first tested at a single concentration of 9524 pg/µL, and, if toxic, a full dose-response curve was generated by testing a serial dilution 1:2 of 8 concentrations. For *Gambierdiscus* spp. cultures, a range of 0.37 to 47.6 pg/µL of dry extract was tested for NHA4, NHA9, NHA14, and NHA22, and a range of 372 to 47,619 of dry extract was tested for NHA1 and NHA24. For *T. gratilla* fractions, the ranges were 186 to 23,809 and 74 to 9524 pg/µL of dry extract for HF50/50 and HF70/30 and HF90/10 and HF100, respectively, and 46.5 to 5952 and 5.8 to 744 pg/µL of dry extract for LF70/30, and LF90/10, and LF100. Each concentration was tested in OV^−^ and OV^+^ conditions, in triplicate per plate, in 2 to 4 independent experiments.

Following another 20–22 h incubation period, cell viability was assessed using 3-(4,5-dimethylthiazol-2-yl)-2,5-diphenyl tetrazolium bromide (MTT) assay. The incubation medium was removed and 60 µL of RPMI-1640 medium containing 0.8 mg/mL of MTT was added to each well. The plates were incubated for 45 min at 37 °C. Finally, the MTT was discarded and 100 µL of dimethyl sulfoxide (DMSO) was added to each well to dissolve the formazan. 

Absorbance was measured at 570 nm on a plate reader (iMark Microplate Absorbance Reader, BioRad, Marnes la Coquette, France). For all experiments, absorbance values of OV^−^ and OV^+^ control wells were around 1, corresponding to 100% viability. Absorbance data were fitted to a sigmoidal dose-response curve (variable slope) based on the 4-parameter logistic model (4PL), allowing the calculation of EC_50_ values using Prism v6.0.7 software (GraphPad, San Diego, CA, USA). Since raw results for all extracts were obtained in pg/µL of dry extract, the EC_50_ values for *T. gratilla* and *Gambierdiscus* samples were further expressed in µg tissue equiv./µL and in cell equiv./µL, respectively.

The toxin content (T) in the extracts was estimated using the formula T = (P-CTX-3C EC_50_/sample EC_50_) and was expressed in ng P-CTX-3C equiv./g wet weight of tissue for *T. gratilla* and in pg P-CTX-3C equiv./cell for *Gambierdiscus* samples. The limit of detection (LOD) was estimated according to the method of Caillaud et al., 2012 [70] and was 0.17 fg P-CTX-3C equiv./cell and 0.02 ng P-CTX-3C equiv./g wet weight of tissue for *Gambierdiscus* and *T. gratilla*, respectively. 

The European Food Safety Authority (EFSA) developed toxicity equivalency factors (TEFs) for individual P-CTXs based on the acute intraperitoneal injection lethal dose (LD_50_) in mice: P-CTX-1 = 1, P-CTX-2 = 0.3, P-CTX-3 = 0.3, P-CTX-3C = 0.2, 2,3-dihydroxy PCTX-3C = 0.1, 51-hydroxy P-CTX-3C = 1, P-CTX-4A = 0.1, P-CTX-4B = 0.05, which can be applied to express individual analogs identified with quantitative detection methods as P-CTX-1B equivalents [28]. Therefore, to enable general comparisons with other published toxicity measurements, the ciguatoxicity measured here (expressed as P-CTX-3C equiv./cell or P-CTX-3C equiv./g wet weight of tissue) could be converted to P-CTX-1B equivalents using the corresponding TEF.

### 4.6. Liquid Chromatography Coupled with Tandem Mass Spectrometry

Liquid chromatography coupled with tandem mass spectrometry (LC-MS/MS) analyses were conducted on freeze-dried samples of *T. gratilla* specimens (whole tissues) collected in July 2015 and November 2016.

Samples were extracted as follows: a homogenate of freeze-dried tissue was extracted twice with acetone. After centrifugation, the supernatants were pooled and evaporated by rotary evaporation. The dry extract was dissolved in aqueous methanol (90:10) and a liquid-liquid partition with hexane was carried out. The 90% methanolic fraction was evaporated and then dissolved in ethyl acetate/methanol (90/10). Then, the remaining extract was purified on silica cartridge solid phase extraction (SPE Si) type Florosil^®^ (Waters, Saint-Quentin, France), and the resulting fraction (SPE Si extract) evaporated under nitrogen and resuspended in 100% methanol prior to LC-MS/MS analysis. (Sciex, Kenwood, CA, USA).

All these fractions or extracts were analyzed by LC-MS/MS in multireaction monitoring (MRM) mode on a triple quadrupole API4000 QTrap (Sciex). P-CTX and biological sample analyses were carried out using the LC-MS/MS method adapted from Yogi et al., 2011 [71]. The instrument used was an LC system (UFLC XR Nexera, Shimadzu, Kyoto, Japan) coupled to a hybrid triple quadrupole-linear ion trap mass spectrometer (API-4000Qtrap, Sciex) equipped with a turboV^®^ ion spray interface. A 1.8 µm C_18_ Zorbax Eclipse plus column (50 × 2.1 mm, Agilent Technologies, Santa Clara, CA, USA) was employed at 40 °C and eluted at 400 µL/min with a linear gradient. Eluent A was water and eluent B was methanol, both containing 2 mM ammonium formate and 50 mM formic acid. The elution gradient ran from 78 to 88% over 10 min and was held for 4 min before re-equilibration for 5 min.

Mass spectrometry detection was operated in positive mode and using MRM (Analyst software, Sciex). The pseudomolecular ions (M+NH_4_)^+^ and (M+H)^+^ were selected as precursor ions. The ions resulting in the successive losses of NH_4_ and/or water molecules were selected as product ions (see Darius et al., 2018 [21]). The MRM experiments were established using the following source settings: curtain gas set at 25, ion spray at 5500 V, turbogas temperature of 300 °C, gas 1 set at 40 and gas 2 set at 60 psi with an entrance potential of 10 V, and declustering potential of 105 V.

Data processing and analysis were achieved with Analyst software (Sciex). Quantification was performed from linear calibration curves generated from P-CTX-3C standard (Wako Chemicals GmbH, Neuss, Germany). To complete chromatogram profiles, a mix of standards (P-CTX-1B, P-CTX-3B, P-CTX-4A, P-CTX-4B, M-seco P-CTX-3C, and 51-OH P-CTX-3C) provided by Louis Malardé Institute (Tahiti, French Polynesia) was injected in the sequence. As for CBA-N2a results, toxin quantification expressed as P-CTX-3C equiv./g wet weight of tissue could be converted to P-CTX-1B equivalents using the corresponding TEF.

In addition to detection of P-CTXs, the presence of other marine biotoxins was investigated according to methods previously described [59,72,73,74]: neurologic shellfish toxins (NSP), i.e., brevetoxins (PbTX1 to PbTX10) and maitotoxins (MTX1 to MTX4); paralytic shellfish poisoning (PSP) toxins, i.e., carbamates (STX, NEO-STX, GTX1-GTX4), *N*-sulfocarbamoyl (GTX5, GTX6, and C1 to C4), and decarbamoyls (dcSTX, dcNEO, dcGTX1-dcGTX4); diarrhetic shellfish poisoning (DSP) toxins, i.e., okadaic acid (OA), dinophysistoxins (DTXs), pectenotoxins (PTXs), azaspiracids (AZAs), and yessotoxins (YTXs); and cyclic imines (fast action toxins, FATs), gymnodimins (GYMs), spirolids (SPXs), and pinnatoxins (PnTXs). 

## Figures and Tables

**Table 1:** Clinical manifestations for the three patients observed 9 days (patients 1 and 2) and 5 days (patient 3) after consuming *Tripneustes gratilla* on Nuku Hiva Island (Marquesas archipelago, French Polynesia) in April and July 2015.

SYMPTOMS ^1^	Occurrence		
	April		July
Patient no.	1	2	3
Symptoms onset (h)	<1	<1	<1
GASTROINTESTINAL			
Nausea/vomiting	x	x	x
Diarrhea	x	x	x
NEUROLOGICAL			
Cold allodynia	x	x	x
Tingling	x	x	x
Touch disturbances	x	x	x
Itching	x	x	x
Burning sensation (throat, mouth)	x	x	x
Headache, dizziness	x	x	x
Vision disorder	x		
OTHER			
Asthenia	x	x	x
Myalgia	x	x	x
Urogenital discomfort/urogenital burning/urogenital pain	x	x	x
Hypothermia, chills	x	x	
Arthralgia			x

^1^ Cardiovascular symptoms were not recorded.

**Table 2:** Cell densities of several benthic dinoflagellate genera found on macroalgal substrates versus window screens (artificial substrates) in Anaho Bay (Nuku Hiva Island, Marquesas archipelago) in July 2015.

Dinoflagellates	D_MA_ *n* = 6 (CV)	D_WS_ *n* = 6 (CV)
*Gambierdiscus*	567 ± 546 (0.96)	14,417 ± 12,280 (0.85)
*Ostreopsis*	3 ± 1 (0.33)	7283 ± 13,726 (1.88)
*Prorocentrum*	32 ± 11 (0.34)	2500 ± 3176 (1.27)

D_MA_ = cell density expressed in number of cells/g (wet weight) of macroalgae; D_WS_ = cell density per WS expressed in cells/150 cm^2^; CV = coefficient of variation.

**Table 3:** *Gambierdiscus* spp. identification of in vitro culture strains isolated from window-screen samples deployed in Anaho Bay (Nuku Hiva Island, Marquesas archipelago).

*Gambierdiscus* Species	*n*	Names of Strains
*G. polynesiensis*	4	NHA-4, -9, -14, -22
*G. carpenteri*	2	NHA-19, -20
*G. pacificus*	2	NHA-1, -24

*n* = number of cultured strains established in the laboratory.

**Table 4:** EC_50_ values and toxin content estimates from eight clonal cultures of *Gambierdiscus* established from Anaho Bay. ND = not detected.

*Gambierdiscus* Species	Strain	EC_50_ Cell Equiv./µL	pg P-CTX-3C Equiv./Cell
*G. polynesiensis*	NHA4	(8.96 ± 0.48) × 10^−4^	1.61 ± 0.08
	NHA9	(6.77 ± 0.87) × 10^−4^	2.13 ± 0.12
	NHA14	(8.61 ± 0.52) × 10^−4^	1.68 ± 0.10
	NHA22	(8.51 ± 0.29) × 10^−4^	1.69 ± 0.06
*G. carpenteri*	NHA19	ND	ND
	NHA20	ND	ND
*G. pacificus*	NHA1	2.66 ± 0.21	(5.44 ± 0.42) × 10^−4^
	NHA24	1.30 ± 0.04	(11.11 ± 0.37) × 10^−4^

**Table 5:** Toxin content estimates in *Tripneustes gratilla* samples collected from Anaho Bay in July 2015 and November 2016, based on EC_50_ values obtained using CBA-N2a. Each value represents the mean ± SD of two to four independent experiments.

Fractions	Toxin Content ^1^	
	July 2015	November 2016
LF70/30	0.38 ± 0.08	ND
LF90/10	17.36 ± 2.37	0.32 ± 0.07
LF100	1.39 ± 0.21	ND
HF50/50	ND	ND
HF70/30	0.11 ± 0.03	ND
HF90/10	0.73 ± 0.21	ND
HF100	0.23 ± 0.05	ND
Total toxin content	20.19 ± 2.96	0.32 ± 0.07

^1^ Results expressed in ng P-CTX-3C equiv./g of tissue.

**Table 6:** Estimation of the relative concentrations of P-CTX congeners in *T. gratilla* collected in Anaho Bay using LC-MS/MS.

Date	P-CTX-3B	P-CTX-3C	51-OH-P-CTX-3C	P-CTX-4A	P-CTX-4B	Total
July 2015	9.57	4.23	5.48	1.16	1.1	**21.5**
November 2016	0.70	0.78	<LD	<LD	<LD	**1.48**

Results expressed in ng P-CTX-3C equiv./g of tissue, with a detection limit of 0.05 ng P-CTX-3C equiv./g of tissue.

**Table 7:** Morphological features of *Tripneustes gratilla* samples.

Island	Area	Date	n	Diameter (mm)	Weight (g)
Nuku Hiva	Anaho	July 2015	24	(70–120) ^1^	32.27 ^2^
		November 2016	32	81.7 ± 9.9	31.4 ± 8.4

^1^ Only min and max values available. ^2^ Weight estimated from diameters and weights measured in 2016.
